# Critical scaling of a two-orbital topological model with extended neighboring couplings

**DOI:** 10.1038/s41598-024-54946-5

**Published:** 2024-02-24

**Authors:** Y. R. Kartik, Ranjith R. Kumar, Sujit Sarkar

**Affiliations:** 1 Theoretical Sciences Division, Poornaprajna Institute of Scientific Research, Bidalur, Bengaluru, 562164 India; 2 Graduate Studies, Manipal Academy of Higher Education, Madhava Nagar, Manipal, 576104 India

**Keywords:** Physics, Condensed-matter physics, Phase transitions and critical phenomena

## Abstract

Extended-range models are the interesting systems, which has been widely used to understand the non-local properties of the fermions at quantum scale. We aim to study the interplay between criticality and extended range couplings under various symmetry constraints. Here, we consider a two orbital Bernevig–Hughes–Zhang model in one dimension with longer (finite neighbor) and long-range (infinite neighbor) couplings. We study the behavior of model using scaling laws and universality class for models with Hermitian, parity-time ($\mathscr{P}\mathscr{T}$) symmetric and broken time-reversal symmetries. We observe the interesting results on multi-criticalities, where the universality class of critical exponent is different than the normal criticalities. Also, the results can be generalized by considering the interplay between criticalities and different symmetry classes of Hamiltonian. Also, with the introduction of extended-range of coupling, there occurs different criticalities, and we provide the analogy to characterize their universality classes. We also show the violation of Lorentz invariance at multi-criticalities and evaluation of short-range limit in long-range models as the highlights of this work.

## Introduction

Topological quantum phase transitions (TQPT) are the milestones in the theory of condensed matter physics due to their distinctive property which can not be apprehended by the Landau theory of symmetry breaking^1,2^. The state lacks order parameter, hence geometric phase (topological invariant/winding number) is considered for topological characterization, where each topological phase is protected by a bulk gap^3,4^. These phases are associated with a pair of localized edge modes, which are nothing but the quasi-particle excitation with a fixed localization (characteristic) length. The TQPT occurs through the vanishing of bulk gap, at which the geometric phase is ill-defined^5,6^.

In general, TQPTs are the second order quantum phase transitions, in which the characteristic length diverges as the system drives towards criticality^7^. This kind of non-analyticities give the idea of scaling laws near the criticality, where the set of critical exponents yield the universality class^7^. In the context of criticality, the study of topological state of matter becomes important as it is the platform for the emergence of exotic particles, unlike fermions and bosons. There are a number of examples which signals the emergence of Majorana zero modes^8^, massive edge modes^9^, and chiral edge modes in the topological systems^10^. Under this scenario, the area has become interesting both from experimental and theoretical perspective.

With the introduction of long-range effect through the coupling parameters, the area became more fertile both at and out of equilibrium conditions^11,12^. Due to the long-range effect, the fermion exhibits its non-local nature, which results in the emergence of massive Dirac^9^ edge modes and violation of area law of von Neumann entropy^13^. There are observations of breakdown of conformal symmetry in long-range models and study of effective field theory to understand the effective Lorentz invariance^14^. The long-range nature can even change the effective dimension of the system^15,16^, as well as can create a transition between two topological phases without gap closing in case of topological systems^17^. Long-range models also a field of curiosity from the perspective of bulk-boundary correspondence^18^, simulation of superconducting circuits^19^, quantum information propagation^20^ and topology at finite temperature^21^.

Topological properties are protected by certain set of symmetries which is called ten fold symmetry classification^22^. These symmetries are responsible for the topological properties such as geometric phase, localization and criticality^5^. This conventional understanding of symmetries modifies with the introduction of the non-Hermiticity. The ten-fold symmetry classification ($\mathscr{A}\mathscr{Z}$) modifies into 38-fold classification ($\mathscr{A}\mathscr{Z}^{\dagger }$), which constitutes the non-Hermitian periodic table of symmetries^23^. The breaking of certain symmetries results in the variation in the topological properties of the systems.

As a generalized platform for our analysis, we adopt a one dimensional version of a two orbital Bernevig–Hughes–Zhang (BHZ) model^24^. Originally, BHZ model is an efficient proposal for the realization of quantum spin Hall effect in two dimension^25^. Here, $\textbf{Z}_2$ topological insulator is realized in a quantum where HgTe is sandwiched between crystals of CdTe. There are numerous works which efficiently explains the topological properties of model both experimentally and theoretically^2,5^. Here, we consider the one dimensional reduced BHZ model to explain the interplay of symmetry and criticality with the extended-range of coupling, and also to characterize the topological criticalities especially when two criticalities meet each other, i.e., multi-criticalities.

## Model Hamiltonian

We consider BHZ model in 1D with extended-range of couplings. The model consists of spinless non-interacting fermions in the s and $p_x$ orbitals, where the interactions like $p_x\pm ip_y$ and the intra-orbital hopping i.e. hopping between $s\longleftrightarrow p_x$ of same unit cells are excluded. The generalized 1D BHZ Hamiltonian is given by^24^

1 $$\begin{aligned} H_0= & {} \sum _{j=1}^{L}\sum _{l=1}^{r}\left( \epsilon _s s_j^{\dagger }s_j+\epsilon _p p_j^{\dagger }p_j-\frac{t_{ps}}{l^{\alpha }}s_j^{\dagger }p_{j-l} \right) +\sum _{j=1}^{L-l}\sum _{l=1}^{r}\left( -\frac{t_s}{l^{\alpha }}s_j^{\dagger }s_{j+l}+\frac{t_p}{l^{\alpha }}p_j^{\dagger }p_{j+l}+\frac{t_{ps}}{l^{\alpha }}s_j^{\dagger }p_{j+l}\right) +H.c. \end{aligned}$$

**Figure 1:**
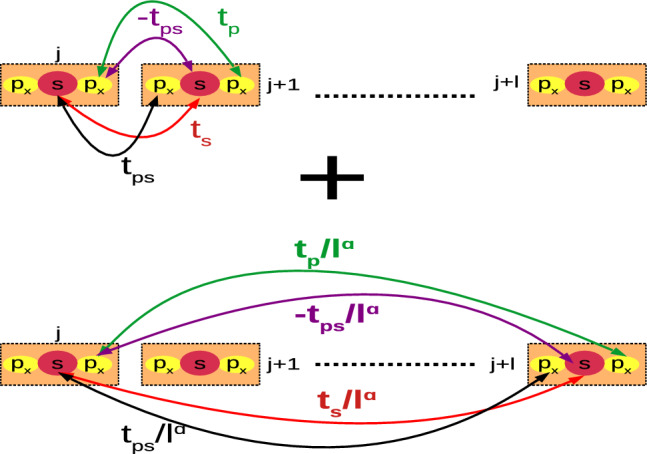
Schematic representation non-interacting extended-range BHZ model in one dimension. Colored rectangles represent the unit cell, with red (yellow) circles representing *s* ($p_x$) orbitals. The neighboring coupling decays with power law for the *l*th neighbor.

The hopping strength between $s_{j}\longleftrightarrow p_{j+1}(s_{j+1}\longleftrightarrow p_j)$ and $s_{j}\longleftrightarrow s_{j+1}(p_{j}\longleftrightarrow p_{j+1})$ is $t_{ps}(-t_{ps})$ and $t_p(t_s)$ respectively. Also, the terms *s* and $p_x$ orbital consists of on site potentials $\epsilon _s$ and, $\epsilon _p$ respectively. Here the term *j* symbolizes the lattice site, which can take a larger value *L*. If the coupling occurs only among the nearest neighbors, it is referred as short-range, whereas, if it occurs among *i*th and $i+l$th sites, it is called extended-range coupling^17,26^ (Fig. 1).

After Fourier transformation, the model can be written in the Bloch Hamiltonian as,

2 $$\begin{aligned} h_0(k)=\frac{1}{L}\sum _{k=1}^{L}\left( \begin{matrix} s_k^{\dagger }&\,&p_k^{\dagger } \end{matrix}\right) H(k) \left( \begin{matrix} s_k\\ p_k \end{matrix}\right) \end{aligned}$$

with

3 $$\begin{aligned} H(k)=\chi _j. \sigma _j. \end{aligned}$$

with $j=0,1,2,3$. Here $\sigma _0,\sigma _{x,y,z}$ and $\chi _{0,x,y,z}$ are identity matrix, Pauli spin matrices and winding vectors respectively. Before studying the system in detail, we need to understand the Hermitian behavior of the model. For this purpose, we substitute $\epsilon _p=-\epsilon _s=\epsilon$ and $t_s=t_s^*=t_p=t_p^*=t$ into Eq. (1). Under standard non-interacting conditions, the Hamiltonian becomes

4 $$\begin{aligned} H(k)=\left( \begin{matrix} \chi _z&{}&{}i\chi _y\\ -i\chi _y&{}&{}-\chi _z \end{matrix} \right) .\end{aligned}$$

with

$$\begin{aligned} \chi _z= & {} -\epsilon -2t\sum _{l=1}^{r}\frac{\cos (kl)}{l^{\alpha }},\hspace{1cm} \chi _y=2t_{ps}\sum _{l=1}^{r}\frac{\sin (kl)}{l^{\alpha }}. \end{aligned}$$

It is to be noted that for $r\rightarrow \infty$ the series involving $\frac{\cos (kl)}{l^{\alpha }}$ and $\frac{\sin (kl)}{l^{\alpha }}$ terms give rise to polylogarithmic functions^11,26^. Here, at first we consider finite number of interacting neighbors (*r*) and analyze the scaling properties through momentum space characterization. The model is called as isotropic, as the coupling parameters decay with the same strength (i.e., $t=t_{ps}$).

Basically, the symmetric properties of the models are unaltered with the addition of extended interacting neighbors. The tenfold classification includes discrete symmetries such as time reversal symmetry (TRS), particle-hole symmetry (PHS) and chiral symmetries (CS). The TRS is an anti-unitary operator, represented as the product of unitary ($\mathscr {U}$) and complex conjugation ($\mathscr {K}$), i.e., $\mathscr {T}=\mathscr{U}\mathscr{K}$. For the spinless systems ,$\mathscr {T}^2=1$ and symmetry is said to be obeyed if the Hamiltonian commutes with the operator, i.e., $\left[ \mathscr {T},H \right] =0$. PHS symmetry is another non-unitary operator with $\mathscr {C}^2=1$ which anti commutes with the Hamiltonian as $\lbrace \mathscr {C},H \rbrace =0$. This signals the transformation between electron and holes under the given range of energy. The product of TRS and PHS gives the CS, which is equivalent to the sub-lattice symmetry in the Hermitian systems. This is an anti-unitary operator with $\lbrace \mathscr {S},H \rbrace =-H$, which reveals the properties of a symmetric spectrum. The general energy spectrum is given by

5 $$\begin{aligned} E_k=\pm \sqrt{(\chi _z(k))^2+(\chi _y(k))^2}. \end{aligned}$$

The topological invariant equation is given by

6 $$\begin{aligned} W=\left( \frac{1}{2\pi }\right) \int _{-\pi }^{\pi }\frac{d}{dk}\tan ^{-1}\left( \frac{\chi _y}{\chi _z}\right) dk, \end{aligned}$$

which yields integer for topological state and $W=0$ for trivial states respectively^27^. Here we calculate different critical exponents using momentum space characterization to understand different criticalities. The universality class can be constructed using different exponents such as dynamic (*z*), localization ($\nu$), crossover (*y*), susceptibility ($\gamma$) and canonical ($\alpha ^*$) critical exponents as bellow.

*Dynamical and crossover critical exponent:* This critical exponent can be calculated by expanding Eq. (5) around the gap closing points $k_0$ as,

7 $$\begin{aligned} E(k,\textbf{g})= & {} \sqrt{(\textbf{g}-\textbf{g}_c)^{2y}+A(k-k_0)^{2z}}, \end{aligned}$$

where $(\textbf{g}-\textbf{g}_c)^{2y}$ signals the distance in the parameter space, *y* is the crossover and *z* is the dynamical critical exponents respectively. Here *y* and *z* determine the gap opening/closing and nature of dispersion, which can be calculated as

8 $$\begin{aligned} E(k\rightarrow k_0,\textbf{g}=\textbf{g}_c)\propto & {} k^z,\hspace{2cm} E(k=k_0,\textbf{g}\rightarrow \textbf{g}_c)\propto \left| \textbf{g}-\textbf{g}_c\right| ^{y}, \end{aligned}$$

*Localization critical exponent:* The topological phase possesses the localization of zero energy edge modes, which are protected by the topology of bulk Bloch electronic states. In the open boundary condition, the Bloch Hamiltonian (Eq. 4) can be written as

9 $$\begin{aligned} \left( \begin{array}{lll} \hat{h}&{}&{}\hat{\Delta }\\ -\hat{\Delta }&{}&{}-\hat{h}\\ \end{array} \right) \left( \begin{array}{lll} \vec {u_n}\\ \vec {v_n} \end{array} \right) =E_n\left( \begin{array}{lll} \vec {u_n}\\ \vec {v_n} \end{array} \right) , \end{aligned}$$

with

$$\begin{aligned} \hat{h}_{ij}= & {} \sum _{l=1}^{r}\frac{t}{l^{\alpha }}(\delta _{j,i+l}+\delta _{j,i-l})+\epsilon \delta _{i,j}\\ \hat{\Delta }_{ij}= & {} \sum _{l=1}^{r}\frac{t_{ps}}{l^{\alpha }}(\delta _{j,i+l}-\delta _{j,i-l}). \end{aligned}$$

Here $\hat{u}_n=\left( u_n(1),u_n(2),\ldots u_n(N)\right)$ and follows the Hermiticity and time reversal symmetry through the relation $u_n=u_n^*$ and $v_n=v_n^*$. Here *n* is the lattice index and the Fourier transformation gives the form $\sum _{k}\psi _k^{\dagger }H(k)\psi _k$ which can also be written in the form of Eq. (4). The ratio of amplitudes of the nth localized Eigen state ($\psi _n$) to the first ($\psi _0$) is given by^28^,

10 $$\begin{aligned} \delta \psi _n=\frac{\psi _n(E=0)}{\psi _1(E=0)}=\left| \frac{\delta g}{A_{2,4}}\right| ^{n-1}. \end{aligned}$$

These zero energy states are localized at the edge of the chain which are ensured by the condition $e^{ik_0}=-\left( \frac{\delta g}{A_{2,4}} \right)$. For zero energy state, $k_0$ is the complex number which leads to $\psi _n=e^{i k_0(n-1)}$, where *n* is the system size. Through Eq. (7), we can calculate $k_0$ as,

11 $$\begin{aligned} k_0=i\left( \frac{\delta g}{A_{2,4}}\right) , \end{aligned}$$

where the dominating term among $A_4$ and $A_2$ decides the value of $k_0$, which finally leads to

12 $$\begin{aligned} \delta \psi _n=e^{\frac{-(n-1)}{\xi }}, \end{aligned}$$

where $\xi =\frac{A_2,4}{|\delta g|^{\nu }}\rightarrow \xi \propto |\delta g|^{-\nu }$, where $\nu$ is the localization critical exponent.

*Susceptibility critical exponent* $\gamma$: This critical exponent can be extracted by the integrand of Eq. (6) which shows divergence as one approaches criticality^27,29–31^. This is also called curvature function (CF), and can be written in the Ornstein-Zernike form (which is traditionally famous for relating different correlation factors with each other).

13 $$\begin{aligned} F(k,\textbf{g})=\frac{\chi _z\partial \chi _y-\chi _y\partial \chi _z}{\chi _z^2+\chi _y^2}. \end{aligned}$$

Here we characterize the critical point as high symmetry (HSP) and non-high symmetry points (non-HSP) based on the behavior of CF around the gap-closing point $k_0$. If the CF exhibits a symmetric nature of gap-closing in the Brillouin zone ($k_0=-k_0$), then it is called HSPs. In such case CF acts as an even function, i.e., $F(k_0+\delta k,\textbf{g})=F(k_0-\delta k,\textbf{g})$ around $k_0$ (Here $\textbf{g}$ is the set of parameters). As we approach the critical point from one side ($\textbf{g}_+\rightarrow \textbf{g}_c$), the CF shows a Lorentzian peak around $k_0$ and flips as we pass criticality ($\textbf{g}_c\rightarrow \textbf{g}_-$). The Lorentzian peak becomes non-analytic at $k=k_0$ which results in the ill-defined winding number at criticality. On the other hand, non-HSP are the points which do not exhibit even nature around $k_0$ but shows non-analytic Lorentzian peak at $k_0$^17^. To obtain the critical exponents, we expand the CF around gap closing points,

14 $$\begin{aligned} F(k,g)\mid _{k=k_0}= & {} \frac{F(k_0,\delta g)}{1+\xi ^2\delta k^2+\xi ^4\delta k^4}, \end{aligned}$$

where the coefficients of $\delta k^2$ and $\delta k^4$ decide the nature of correlation ($\xi$). For our 1D system, we consider Berry connection as our CF, whose behavior is characterized by the critical exponents $\nu$ and $\gamma$. i.e.^27,32^,

15 $$\begin{aligned} F(k_0,\textbf{g})\propto |\textbf{g}-\textbf{g}_c|^{-\gamma },\xi \propto |\textbf{g}-\textbf{g}_c|^{-\nu }. \end{aligned}$$

Here $\gamma$ and $\nu$ are the susceptibility and localization critical exponent, which signal the exponent associated with the Berry connection^33^.

*Ground state energy:* For a system with real energy spectrum, each energy interval *dE* contains $|k|^d$ occupied states, where *d* is the spacial dimension of the system. The ground state energy (GSE) indicates TQPTs with the singularities in the parameter space ($\omega _{singular}$), where the order of the its derivative is related to the order of transition. Thus a relation can be established between GSE and other critical exponents as,

16 $$\begin{aligned} \omega _{singular}\propto & {} |\delta g|^{\nu (d+z)} \end{aligned}$$

where $(d+z)$ is the effective dimension^34^. This concept leads to the famous relation

17 $$\begin{aligned} 2-\alpha ^*=\nu (d+z). \end{aligned}$$

known as Josephson’s hyper-scaling relation which relates the effective dimensionality with the order of phase transition^7^. (Here $\alpha ^*$ is called canonical critical exponent and not to be confused with decay parameter $\alpha$). To analyze the order of transition, we perform the scaling of the singular part of the GSE^34^. As the GSE is sensitive to system size, it needs a multiplication of every length of GSE by localization factor^34^i.e.,

18 $$\begin{aligned} \omega _{singular}\propto g^{2-\alpha }g^{-\nu }, \end{aligned}$$

which gives the effective dimension of the system.

Thus, the set of critical exponents yield the universality class which is an efficient to categorize different criticalities. In the following section, we considers different examples to understand the interplay between criticality and extended range coupling under various symmetry constraints.

## Results

Here we present three different ranges of coupling, i.e., short-range, extended-range and long-range for Hermitian, broken TRS and $\mathscr{P}\mathscr{T}$ symmetric respectively.

### Hermitian condition

Under the standard Hermitian conditions, we obtain the Hamiltonian,

19 $$\begin{aligned} H_1(k)=\left( \begin{matrix} -\epsilon -\sum _{l=1}^{r}\frac{2t}{l^{\alpha }}\cos (kl)&{}&{}\sum _{l=1}^{r}2i\frac{t_{ps}}{l^{\alpha }}\sin (kl)\\ \\ -\sum _{l=1}^{r}2i\frac{t_{ps}}{l^{\alpha }}\sin (kl)&{}&{}\epsilon _p+\sum _{l=1}^{r}\frac{2t}{l^{\alpha }}\cos (kl) \end{matrix} \right) .\end{aligned}$$

Here the Hamiltonian follows TRS, PHS and CS as shown in Table 1, which results in AIII symmetry class of AZ classification. In general, when the Hamiltonian obeys TRS, PHS and CS symmetries, a topological superconductor falls under BDI class and makes a 1D model as a *Z* topological invariant . But in a topological insulator (like SSH), the chiral symmetry is enforced and other symmetries are accidental. This is just a matter of choice and does not alter the physics of criticality. (This factor becomes significant, when one prefers to characterize the topology of the system purely based on the symmetric behavior. Here we just restrict ourselves for the critical characterization).

**Table 1:** Symmetry operation of a Hermitian model.

Symmetry	Operation	Result
TRS	$\mathscr {T}H(k)\mathscr {T}^{-1}=H(-k)$	$\checkmark$
PHS	$\mathscr {C}H(k)\mathscr {C}^{-1}=-H(-k)$	$\checkmark$
CS	$\mathscr{T}\mathscr{C}H(k)\mathscr {T}^{-1}\mathscr {C}^{-1}=-H(-k)$	$\checkmark$

Here TRS, PHS and CS represent time-reversal, particle-hole and chiral symmetries respectively.

The operators $\mathscr {T}$ is nothing other than the complex conjugate $\mathscr {K}$. The operator $\mathscr {C}$ is the combination of two terms, i.e., $\mathscr {C}=U_c\mathscr {K}$. Here $U_c$ is a unitary matrix and for the current case it yields $\sigma _x$. The operator $\mathscr{T}\mathscr{C}$ is the combination of above operators. Here, we consider three different ranges of coupling to understand the behavior of criticality, i.e., short-range, extended-range and long-range.

#### Short-range

As a first case, we consider the coupling up to first interacting neighbor only, i.e. short-range ($\alpha \rightarrow \infty$). The winding vectors are given by

20 $$\begin{aligned} \chi _z= & {} -\epsilon -2t\cos (k),\hspace{2cm} \chi _y=2t_{ps}\sin (k) \end{aligned}$$

**Figure 2:**
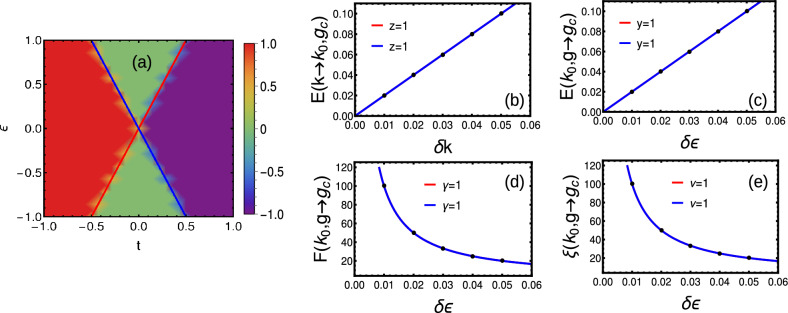
(**a**) Phase diagram of short-range model with $t_{ps}=1$. (**b**–**e**) Dynamical, crossover, susceptibility and localization critical exponents around $k=0$ (red) and $\pi$ (blue) respectively.

Here we consider $t_{ps}=1$ for the simplicity. The phase diagram is given by Fig. 2a, where the criticality occurs with linear dispersion at $\epsilon =2t$ and $\epsilon =-2t$ for $k=0$ and $\pi$ respectively (Fig. 2b). Here we obtain topological phase for $\epsilon <2t$ and trivial phase for $\epsilon >2t$.

By using Eqs. (20) and (13), we get CF, which is a HSP around $k=0$ and $\pi$. The GSE shows the singularity for the second order derivative signaling a second order phase transition where the critical exponents are presented in Fig. 2b–e.

#### Extended-range

**Figure 3:**
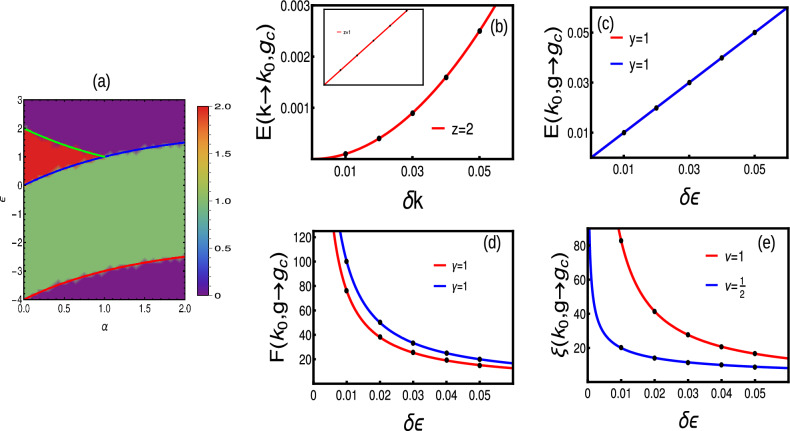
(**a**) Phase diagram of extended-range with two interacting neighbors. (**b**) Dispersion giving $z=2$ at multi-criticality. The inset represents linear dispersion at normal criticalities. (**c**–**e**) crossover, susceptibility and localization critical exponents around $k=\pi$ for multi-criticality (blue) and normal criticality (red) respectively.

We consider a simple extended-range model having second nearest neighbor ($r=2$) coupling with $t=t_{ps}$ condition. The phase diagram is given by Fig. 3a. The quasi-energy dispersion at $k=0$ is given by Eq. (54), which remains linear for all the values of $\alpha$ as the term $A_2=2t \left( 1-\frac{4}{2^{\alpha }}\right)$ always dominates over $A_4$. Thus, at $k=0$, the dynamical critical exponent is $z=1$ irrespective of the value of $\alpha$. Due to the effect of multi-criticality, the nature of dispersion is quadratic ($z=2$) around $k=\pi$ at $\alpha =1$ as the term $A_4=\sqrt{4 \left( \frac{4}{2^{\alpha }}-1\right) t \left( -2 \left( \frac{1}{2^{\alpha }}-1\right) t -\epsilon \right) +\left( 2 \left( \frac{2}{2^{\alpha }}-1\right) t \right) ^2}$ dominates over $A_2$. This is a nice example of the breaking of Lorentz invariance (for $\alpha =1$), the instances of which are the area of curiosity in the condensed matter systems (For analytical calculations, please refer “Method” section). We explain on this more in the following section.

Similarly, the dominating term among *A*2 and *A*4 determines the exponent $\nu$. At $k=0$, the term $A2>A4$, and contributes majorly to the divergence of localization $\xi$ by yielding $\nu =1$ for all the values of $\alpha$. In the vicinity of multi-critical point, the behavior is quite different. At $k=\pi$, the term $A2>A4$ for all values except $\alpha =1$, which gives $\nu =1$ in this regime. At $\alpha =1$ the term $A4>A2$ and yields $\nu =1/2$. Thus, the scaling law $z\nu =2\times 1/2=1$ also remains valid at multi-criticality (Fig. 3b–e).

To understand the order of phase transition, we consider Josephson hyper-scaling relation Eq. (17) as

21 $$\begin{aligned} 2-\alpha ^*= & {} 1(1+1)=2,\hspace{0.5cm}\text {for}\hspace{0.2cm}\alpha \ne 1,\nonumber \\= & {} \frac{1}{2}(1+2)=\frac{3}{2},\hspace{0.5cm}\text {for}\hspace{0.2cm}\alpha =1. \end{aligned}$$

signaling a fractional order of transition at multi-critical points. To understand the nature of scaling, we substitute into Eq. (18), giving

22 $$\begin{aligned} \omega _{singular}\propto & {} g^{2}g^{-1}\propto g^1,\hspace{0.5cm}\text {for}\hspace{0.2cm}\alpha \ne 1,\nonumber \\\propto & {} g^{\frac{3}{2}}g^{-\frac{1}{2}}\propto g^1,\hspace{0.5cm}\text {for}\hspace{0.2cm}\alpha =1. \end{aligned}$$

Thus, the GSE scales similarly for normal criticality and at multi-critical point even though both belong to different universality classes. On the other hand, both at criticality and multi-criticality, the GSE shows non-analyticity for the second order derivatives (not shown here). This shows that, the bulk topological transitions are the second order transitions even at multi-criticality.

At multi-criticality ($k=\pi ,\alpha =1, \epsilon =1$), the two critical lines intersect each other, which separate at least three topological phases. This kind of intersection results in fixed point configuration where the CF fails to exhibit the even nature around the HSP. Thus, at the multi-criticality, CF does not exhibit non- analyticity, instead shows a curve with constant height and varying width^17^. This kind of behavior is absent in other transitions. Interestingly, the scaling of CF gives $\gamma =1$ for all criticalities including multi-criticality (Fig. 3d).

The further increase in the neighbor coupling produces different phase diagram and corresponding critical lines. As one increases the coupling neighbors (for all $r>2$), there occurs a staircase of topological transitions only among even-to-even and odd-to-odd winding numbers^17^. Interestingly, we observe multi-critical behavior, only in the presence of even neighbor couplings. Thus, all the topological transitions (irrespective of neighbors) falls to single universality class except multi-criticality (Table 2).

**Table 2:** A comparison of universality class of critical exponents between the short-range, extended-range and long-range models.

Model	$\hspace{0.2cm}k_0\hspace{0.2cm}$	$\hspace{0.2cm}z\hspace{0.2cm}$	$\hspace{0.2cm}\nu \hspace{0.2cm}$	$\hspace{0.2cm}\gamma \hspace{0.2cm}$	$\hspace{0.2cm} y\hspace{0.2cm}$	$2-\alpha ^*$
Short-range	0	1	1	1	1	2
	$\pi$	1	1	1	1	2
Extended-range (multi-criticality)	0	1	1	1	1	2
	$\pi$	2	1/2	1	1	3/2
Extended-range (normal criticality)	0	1	1	1	1	2
	$\pi$	1	1	1	1	2
Long-range						
$\alpha <1$	0	D	IL	IL	IL	IL
$1<\alpha <2$		$z<1$	–	–	1	H
$\alpha >2$		1	1	1	1	2
$\forall$ $\alpha$	$\pi$	1	1	1	1	2

Here IL, D and H represent ill-defined, divergent and higher order quantities.

#### Long-range

When there are infinite number of neighboring coupling, the pseudo-spin parameters takes polylogarithmic behavior as^11,17^,

23 $$\begin{aligned} \chi _y(k)= & {} 2t\left( \frac{Li_{\alpha }[e^{i k}]-Li_{\alpha }[e^{-i k}]}{2i}\right) ,\hspace{2cm} \chi _z(k)=-\epsilon -2t\left( \frac{Li_{\alpha }[e^{i k}]+Li_{\alpha }[e^{-i k}]}{2}\right) \end{aligned}$$

**Figure 4:**
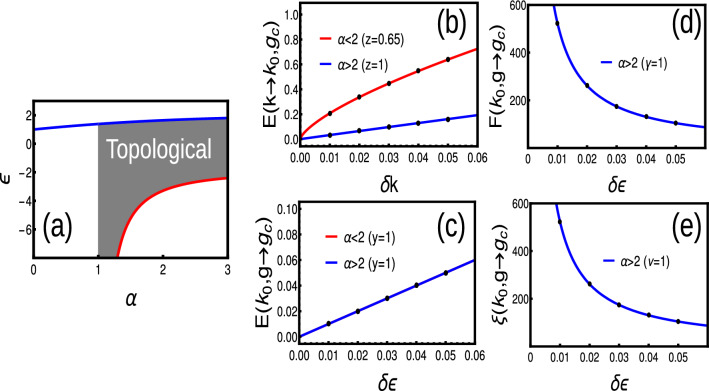
(**a**) Phase diagram of long-range model. (**b**–**e**) Dynamical, susceptibility, crossover and localization critical exponents for Hermitian long-range model.

The energy (Eq. 5) gap closing occurs for $k=0(\alpha >1)$ and $\pi (\forall \alpha )$. There occurs a diverging energy spectrum around $k=0$ for $\alpha \le 1$, due to the polylogarithmic nature of $\chi _y$^11^. Thus, one can get the phase diagram as (Fig. 4a) and the corresponding critical lines are $\epsilon =-2t(2^{1-\alpha }-1)\zeta [\alpha ]$ for $\forall \alpha$ at $k=\pi$ and $\epsilon =-2t Li_{\alpha }[1]$ for $\alpha >1$ at $k=0$ respectively^17,26^. Due to the non-analytical nature of the CF, the topological invariant is ill-defined for $\alpha <1$. However, here the CF shows an removable singularity, where the singularity can be integrated out and the WN yields fractional values (Fig. 4a).

Expansion of polylogarithmic functions is given by^35^,

24 $$\begin{aligned} Li_{\alpha }[e^{ik}]=\Gamma [1-\alpha ](-ik)^{\alpha -1}+\sum _{n=0}^{\infty }\frac{\zeta [\alpha -n]}{n!}(ik)^n \end{aligned}$$

For the detailed study, we can expand the polylogarithmic function around $k=0$ as

25 $$\begin{aligned} \chi _z= & {} -\epsilon -2t( \Gamma [1-\alpha ](k)^{\alpha -1}\sin \left( \frac{\pi \alpha }{2}\right) +\sum _{n=0}^{\infty }\frac{\zeta [\alpha -n]}{n!}(k)^n\cos \left( \frac{\pi n}{2}\right) ),\nonumber \\ \chi _y= & {} 2t(\Gamma [1-\alpha ](k)^{\alpha -1}\cos \left( \frac{\pi \alpha }{2}\right) +\sum _{n=0}^{\infty }\frac{\zeta [\alpha -n]}{n!}(k)^n\sin \left( \frac{\pi n}{2}\right) ). \end{aligned}$$

Thus the pseudo-spin vectors are monitored by the gamma function which depends on $\alpha$. Here, $\alpha$ can influence the properties of the phase diagram, gap closing, energy dispersion and Fermi velocity. The energy dispersion is given by the Eq. (5) with pseudo-spin vectors explained in Eq. (23). To understand the polylogarithmic nature, we express in terms of Eq. (25) and obtain the dispersion as^11^,

26 $$\begin{aligned} E(k,\textbf{M})= & {} \sqrt{Ak^{2\alpha -2}+Bk^{\alpha -1}+C}, \end{aligned}$$

where *A*, *B* and *C* are constants. Thus, as $k\rightarrow 0$ the energy vanishes only for $\alpha >1$ while as $k\rightarrow \pi$, the energy vanishes for all values of $\alpha$. Event though the energy vanishes the derivatives fail to vanish in the limit $k\rightarrow 0$ for $1<\alpha <2$ which reflects in corresponding critical exponent.

By substituting Eq. (25) into Eq. (5) and expanding the energy dispersion equation around the gap closing point $k_0$, up to a leading order, we get Eq. (7) with coefficients

$$\begin{aligned} \delta g= & {} (-\epsilon -2t Li_{\alpha }[\pm 1]),\hspace{0.5cm}A_4=t^2Li_{\alpha -2}[\pm 1],\hspace{0.5cm} A_2=4t^2Li_{\alpha }^2[\pm 1]-t Li_{\alpha -2}[\pm 1](-\epsilon -2t Li_{\alpha }[\pm 1]). \end{aligned}$$

for $k=0$ and $\pi$ respectively. For $k=\pi$, the $A_2$ term dominates for all values of $\alpha$, which ensures a linear dispersion i.e., $E(k_0,\textbf{g}_c)\propto k$. For $k=0$, the $A_2$ term shows a divergence with a factor $\Gamma [1-\alpha ]$. Thus, $\alpha >2$ region shows a linear dispersion with $E(k_0,\textbf{g}_c)\propto k$, while the $1<\alpha <2$ region shows a dispersion with $z<1$ and $y=1$ (Fig. 4b,d). Our results are in agreement with the Ref.^14^, which deals with a similar model through field theoretical methods.

The CF is given by Eq. (13). To understand the $\alpha$ dependent non-analyticity, we substitute Eq. (25) into Eq. (13) which is expanded around $k_0$ the first leading order and after few steps of simplification, we get the CF in Ornstein-Zernike form as

27 $$\begin{aligned} F(k,\textbf{g})\propto \frac{\frac{A k^{\alpha -2}}{B k^{\alpha -1}}-\frac{Ck^{2\alpha -3}}{Dk^{2\alpha -2}}}{1+(\frac{Ek^{\alpha -1}}{Fk^{\alpha -1}})^2} \end{aligned}$$

There are three possible cases,

- When $\alpha <1$, the term $k^{\alpha -2}$ dominates and $F(k,\textbf{g})\rightarrow \infty$ as $k\rightarrow 0$, irrespective of $\textbf{g}\rightarrow \textbf{g}_c$
- When $1<\alpha <2$, the term $k^{\alpha -2}$ dominates and $F(k,\textbf{g})\rightarrow \infty$ as $k\rightarrow 0$, irrespective of $\textbf{g}\rightarrow \textbf{g}_c$
- When $\alpha >2$, again the term $k^{\alpha -2}$ dominates and $F(k,\textbf{g})\rightarrow \infty$ as $k\rightarrow 0$ with $\textbf{g}\rightarrow \textbf{g}_c$

Here we find CF in Ornstein-Zernike form only for $\alpha >2$ around $k=0$ and for all $\alpha$ around $k=\pi$, which yield $\gamma =\nu =1$ in these regions (Fig. 4c,e). Thus, The long-range model attains the universality class of short-range for the range $\alpha >2$, which can be considered as the short-range limit of the model.

### Hermitian case with broken TRS

The Hermitian model preserves the time-reversal symmetry and it can be broken with the introduction of a phase difference in the hopping term as $t_s\rightarrow |t|e^{i\phi }$, where *t* is a real quantity. This results in a complex hopping and the model shifts from AIII to D symmetry class in AZ symmetry classification^26^, where PHS is preserved (Table 3).

**Table 3:** Symmetry operation of a Hermitian model with broken time-reversal symmetry.

Symmetry	Operation	Result
TRS	$\mathscr {T}H(k)\mathscr {T}^{-1}\ne H(-k)$	X
PHS	$\mathscr {C}H(k)\mathscr {C}^{-1}=-H(-k)$	$\checkmark$
CS	$\mathscr{T}\mathscr{C}H(k)\mathscr {T}^{-1}\mathscr {C}^{-1}=-H(-k)$	X

Here TRS, PHS and CS represent time-reversal, particle-hole and chiral symmetries respectively.

The breaking of TRS results in a gapless region along with topological and trivial phases. The analysis shows that $\phi$ takes the values in the regime $\left[ 0,\pi /2\right]$ which produces different phase diagrams^26^. The Hamiltonian is given by,

28 $$\begin{aligned} H_2= & {} \sum _{j=1}^{L}\sum _{l=1}^{r}\left( \epsilon (s_j^{\dagger }s_j+ p_j^{\dagger }p_j)-\frac{t_{ps}}{l^{\alpha }}s_j^{\dagger }p_{j-l} \right) +\sum _{j=1}^{L-l}\sum _{l=1}^{r}\left( -\frac{t}{l^{\alpha }}e^{i\phi }(s_j^{\dagger }s_{j+l}+p_j^{\dagger }p_{j+l})+\frac{t_{ps}}{l^{\alpha }}s_j^{\dagger }p_{j+l}\right) +H.c. \end{aligned}$$

After Fourier transform the Hamiltonian can be written as

29 $$\begin{aligned} H_2(k)=\chi _0\sigma _0+\chi _z\sigma _z+\chi _y\sigma _y \end{aligned}$$

with

30 $$\begin{aligned} \chi _0= & {} \sum _{l=1}^{r}\frac{2t}{l^{\alpha }}\sin (\phi )\sin (kl),\hspace{0.5cm} \chi _z=-\epsilon -\sum _{l=1}^{r}\frac{2t}{l^{\alpha }}\cos (\phi l)\cos (kl),\hspace{0.5cm} \chi _y=\sum _{l=1}^{r}2\frac{t_{ps}}{l^{\alpha }}\sin (kl). \end{aligned}$$

The energy dispersion is given by

31 $$\begin{aligned} E_k= & {} \chi _0\pm \sqrt{\chi _z^2+\chi _y^2}. \end{aligned}$$

The curvature function is not directly involved in defining the topological invariant or number of edge modes. But the topological index can be verified with the presence or absence of the edge modes. The phase diagram consists of gapped and gapless phases which can be determined by the relation^26^

32 $$\begin{aligned} \eta =min_{\lbrace k\rbrace }\lbrace E^+E^-\rbrace max_{\lbrace k\rbrace }\lbrace E^+E^-\rbrace . \end{aligned}$$

The quantity $sign(\eta )=\pm 1$ signals the gapped and gapless phases respectively. The transition from gapped to gapless phase represents the topological transition and the corresponding critical properties can be measured. The topological phase occurs in the regime^26^

33 $$\begin{aligned} -2t\sum _{l=1}^{r}\frac{\cos (\phi )}{l^{\alpha }}<\epsilon <-2t\sum _{l=1}^{r}(-1)^l\frac{\cos (\phi )}{l^{\alpha }}. \end{aligned}$$

When the angle $\phi$ is independent of the *l*, i.e., $\phi _l=\phi$ the relation becomes

34 $$\begin{aligned} -2r\cos (\phi )<\frac{\epsilon }{t}<-2\cos (\phi )\left( \frac{1+(-1)^r}{2} \right) . \end{aligned}$$

We observe two kind of gapless regions here. The gapless region because of the momentum vector ($k=0,\pi$) and the phase factor $\phi$. for a finite value of $\phi$, the energy spectrum yields an elliptic equation $\chi _0^2=\chi _z^2+\chi _y^2$. The model obeys TRS only for $0, \pi$. Hence for all other finite values of $\phi$, the $\chi _0$ term produce a finite value and a 2D elliptic region is produced with gapless spectrum and an ill-defined topological index. This region contains a horizontal boundary (in $t_{ps}-\epsilon$ parameter space) with $t_{ps}=\pm \sin (\phi )$ condition and a vertical boundary with $\epsilon =\pm \cos (\phi )$ condition. As the number of interacting neighbors increase, vertical boundary gets the multiplicative terms. A hemispherical cap is added to the vertical boundaries, which touch the criticality $k=0,\pi$. The region outside this elliptical structure contains integer topological invariant and the criticality $k=0,\pi$ separates topological and trivial phases.

The interface of these two gapless regions constitutes a multi-critical point, which is unique than other criticalities. Thus, we consider three ranges of couplings to understand the behavior of these criticalities. Here, our interest is to explore the behavior of multi-criticality and for our purpose we consider a single parameter space $\phi =3\pi /10$. The further variation of $\phi$ alters the phase diagram^26,36^, but the general behavior of criticalities follow the similar pattern.

#### Short-range

With the coupling up to first nearest neighbor, we write Eq. (30) as

35 $$\begin{aligned} \chi _0= & {} 2t\sin (\phi )\sin (k),\hspace{0.5cm} \chi _z=-2t\cos (\phi )\cos (k)-\epsilon ,\hspace{0.5cm} \chi _y=2t_{ps}\sin (k). \end{aligned}$$

The energy dispersion is given by Eq. (31) and criticality occurs at

36 $$\begin{aligned} (-2t\cos (\phi )\cos (k)-\epsilon )^2+(2t_{ps}\sin (k))^2=(2t\sin (\phi )\sin (k))^2. \end{aligned}$$

The gap closing occurs at $k=0,\pi$ with criticality $\epsilon =\pm 2t\cos (\phi )$. The parameter $\phi$ takes the value between 0 and $\pi /2$ where the horizontal and vertical boundaries are determined by $\epsilon =\pm 2t\cos (\phi )$ and $t_{ps}=\pm t\sin (\phi )$ respectively. The ends of the gapless region touches the points $(t_{ps},\epsilon )=(0,\pm 2)$, which creates an elliptical gapless region as shown in Fig. 9a.

**Figure 5:**
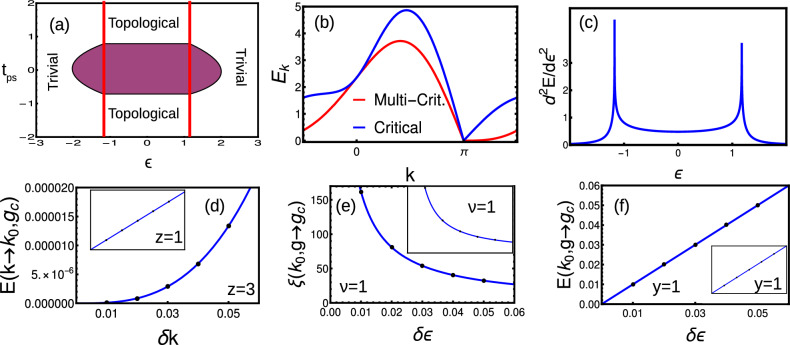
(**a**) Phase diagram of Hermitian model with broken TRS with $\phi =3\pi /10$. The colored gapless region is ill-defined and topological quantities can not be defined in this region. (**b**) Energy dispersion at normal criticality (blue) and multi-criticality (red) respectively. There occurs different dispersion around multi-criticality. (**c**) Ground-state energy density which do not recognizes the spherical cap of the elliptical region. The peaks represent the criticalities $\epsilon =\pm 2t\cos (\phi )$. (**d**–**f**) Dynamical, localization and crossover critical exponent at right side of a multi-critical point. The inset figures represent corresponding critical exponent towards the left side of the multi-critical point.

The energy dispersion shows a linear spectrum at $\epsilon =\pm \cos (\phi )$ while at multi-criticalities show linear dispersion only at one side. (Fig. 5b). The broken TRS leads to the breaking of even nature of spectrum around the gap closing pint. Thus, we get $z=1$ and $z=3$ around left and right sides of gap closing point respectively (Fig. 5d). The crossover critical exponent yields linear curve for both left and right side of the multi-critical point (Fig. 5f). The localization of the edge mode can be determined by Eq. (36), which yield linear spectra around multi-criticality (Fig. 5e).

#### Extended-range

With the coupling up to second nearest neighbor, we write Eq. (30) as

37 $$\begin{aligned} \chi _0= & {} 2t\sin (\phi )\left( \sin (k)+\frac{\sin (2k)}{2^{\alpha }}\right) ,\hspace{0.5cm} \chi _z=-2t\cos (\phi )\left( \cos (k)+\frac{\cos (2k)}{2^{\alpha }}\right) -\epsilon ,\nonumber \\{} & {} \hspace{0.5cm} \chi _y=2t_{ps}\left( \sin (k)+\frac{\sin (2k)}{2^{\alpha }}\right) \end{aligned}$$

The energy dispersion is given by Eq. (31) and criticality occurs at

38 $$\begin{aligned} \left( -2t\cos (\phi )\left( \cos (k)+\frac{\cos (2k)}{2^{\alpha }}\right) -\epsilon \right) ^2+\left( 2t_{ps}\left( \sin (k)+\frac{\sin (2k)}{2^{\alpha }}\right) \right) ^2=\left( 2t\sin (\phi )\left( \sin (k)+\frac{\sin (2k)}{2^{\alpha }}\right) \right) ^2 \end{aligned}$$

**Figure 6:**
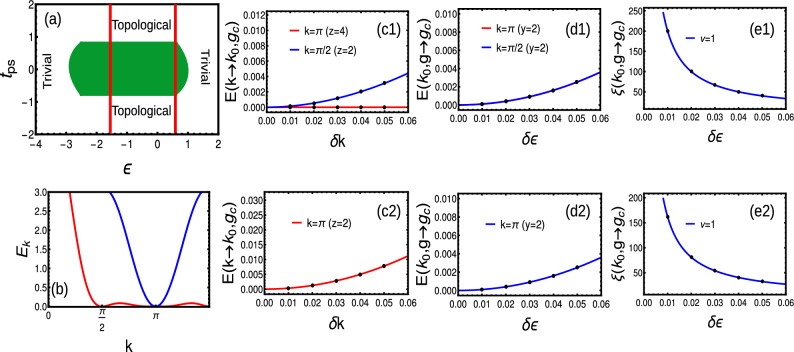
(**a**) Phase diagram of extended-range Hermitian model with broken TRS with $\phi =3\pi /10$. There are two coupling neighbors, with parameter space $t=\alpha =1$. The colored gapless region is ill-defined and topological quantities can not be defined in this region. (**b**) Energy dispersion at normal criticality (blue) and multi-criticality (red) respectively. There occurs different dispersion around Multi-criticality. (**c**) Ground-state energy density which do not recognizes the spherical cap of the elliptical region. The peaks represent the criticalities $\epsilon =\pm 2t\cos (\phi )$. (**d**–**f**) Dynamical, localization and crossover critical exponent at left side of a multi-critical point. The inset figures represent corresponding critical exponent towards the right side of the multi-critical point.

The phase diagram consists of an ill-defined topological region with horizontal and vertical boundaries at $\epsilon =-2t\cos (\phi )(\pm 1+\frac{1}{2^{\alpha }})$ and $t_{ps}=\pm t\sin (\phi )$ respectively. The spherical cap at the ends of the cylinder touches the point $(t_{ps},\epsilon )=(0,-2t(\pm 1+\frac{1}{2^{\alpha }})$. The phase diagram consists of multi-criticality at ($t_{ps}=\pm 0.8090,\epsilon = \pm 0.5877$) which behaves different than other criticalities. Normal criticalities show single gap closing while multi-criticality shows two gap closings at $k=\pi$ and $\pi /2$ respectively (Fig. 6). The normal criticality exhibits quadratic dispersion, while the multi-criticality exhibits dispersion with $z=4$ and 2 respectively (Fig. 6). The crossover and localization critical exponents can be calculated from Eqs. (54) and (54) with $y=\nu =1$.

#### Long-range

Due to the long-range effects, Eq. (30) becomes

39 $$\begin{aligned} \chi _0= & {} 2t\cos (\phi )\left( \frac{Li_{\alpha }[e^{i k}]-Li_{\alpha }[e^{-i k}]}{2i}\right) ,\hspace{0.5cm} \chi _z=-\epsilon -2t\cos (\phi )\left( \frac{Li_{\alpha }[e^{i k}]+Li_{\alpha }[e^{-i k}]}{2}\right) ,\nonumber \\ \chi _y= & {} 2t_{ps}\left( \frac{Li_{\alpha }[e^{i k}]-Li_{\alpha }[e^{-i k}]}{2i}\right) . \end{aligned}$$

**Figure 7:**
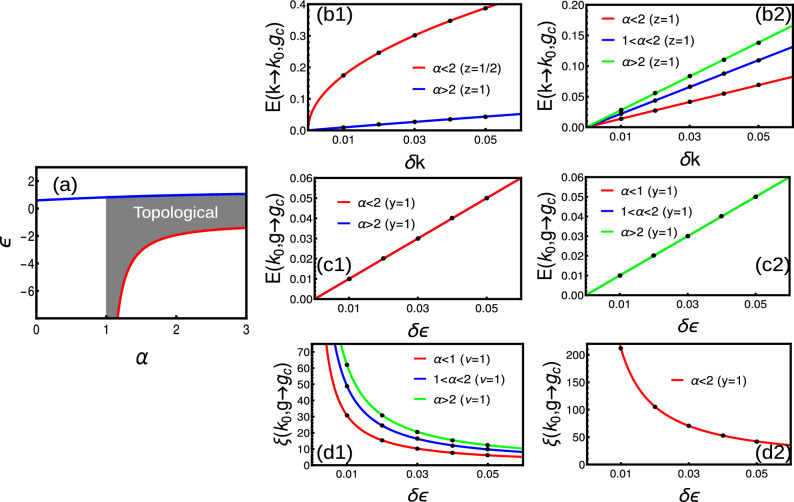
(**a**) Phase diagram of long-range Hermitian model with broken TRS with $t=1,\phi =3\pi /10$. The colored region is the topological phase and $\alpha <1$ is an ill-defined region. (**b**1-**b**2) Dynamical critical exponent at $k=0$ and $k=\pi$ criticalities respectively. (**c**1-**c**2) Crossover critical exponent at $k=0$ and $k=\pi$ criticalities respectively. (**d**1-**d**2) Localization critical exponent at $k=0$ and $k=\pi$ criticalities respectively.

Here we consider the value of $\phi$ independent of index ’*l*’, hence they are not expressed in terms of polylogarithmic functions. The criticalities occur at $\epsilon =-2t\cos (\phi )Li_{\alpha }(\pm 1)$ producing the phase diagram Fig. 7a. The energy dispersion can be obtained by substituting Eq. (39) into Eq. (31) as shown in Fig. 7b,c. At $k=0$, the dispersion is divergent with ill-defined critical exponent. For the range $1<\alpha <2$ and $\alpha >2$, we observe a square root ($z=1/2$) and linear ($z=1$) dispersion respectively (Fig. 7b1). For $k=\pi$, dispersion remains linear for values of $\alpha$ with $z=1$ (Fig. 7b2). The crossover critical exponent remains same $(y=1)$ for all criticalities (Fig. 7c1-c2). The CF can not be expressed in terms of Ornstein-Zernike form, hence the critical exponents $\gamma$ can not be expressed with current methodology. But through the relation Eq. (54), we obtain $\nu =1$ for $\alpha >2$ regime (Fig. 7d1-d2). A comparison of all critical exponents can be given by Table 4

**Table 4:** A comparison of universality class of critical exponents between short, extended and long-range of Hermitian model with broken time-reversal symmetry.

Model	$\hspace{0.2cm}k_0\hspace{0.2cm}$	$\hspace{0.2cm}z\hspace{0.2cm}$	$\hspace{0.2cm}y\hspace{0.2cm}$	$\nu$
Short-range	0	1	1	1
	$\pi$	1	1	1
Extended-range	0	1	1	1
	$\pi$	1	1	1
Multi-criticality		(3,1)	1	1
Long-range				
$\alpha <1$	0	D	IL	IL
$1<\alpha <2$		$z<1$	1	IL
$\alpha >2$		1	1	1
$\forall$ $\alpha$	$\pi$	1	1	1

Here IL and D represent ill-defined and divergent quantities.

### $\mathscr{P}\mathscr{T}$ symmetric non-Hermitian models

In general, the imbalance in the hopping or the addition of complex potential leads to a non-Hermitian behavior of the system, with a complex energy spectrum. With the addition of $\mathscr{P}\mathscr{T}$ symmetry, the space-time reflection can be preserved, where the models can be treated equivalent to Hermitian counterpart irrespective of the non-Hermitian signatures. In our case, non-Hermiticity can be introduced into the model with a non-reciprocity parameter $\delta$ in the hopping term as

40 $$\begin{aligned} H_3(k)=H_1+ \sum _{l=1}^{r}\frac{\delta }{l^{\alpha }}\left( \sum _{i=1}^{N-1}(s_j^{\dagger }p_{j+l}-p_{j+l}^{\dagger }s_j)-\sum _{i=1}^{N}(s_{j+1}^{\dagger }p_{j}-p_{j}^{\dagger }s_{j+1})\right) +H.c. \end{aligned}$$

Thus the forward hopping ($s_j\rightarrow p_{j+l}$) and backward ($s_j\leftarrow p_{j+l}$) becomes $t-\delta$ and $t+\delta$ respectively. Thus, in the region $t_{ps}>|\delta |$ the Hamiltonian produces the real spectra and $t_{ps}<|\delta |$ produces complex energy spectrum respectively. The real part contributes to the filling of Fermi level, while the imaginary part contributes to the phase. Hence, we obtain the information of dynamical and crossover critical exponents from the energy dispersion. Through the analysis of bi-orthonormal vectors, the complex Berry phase can be constructed, and the integrand can provide the information of the curvature function. The curvature function contains the complex form, where the real part contributes to the argument and the imaginary contributes towards amplitude. The non-reciprocity in hopping produces a coalescence of Eigen vectors, which results in non-Hermitian skin effect and breaking of bulk-edge correspondence. However, here we only concentrate on the critical properties under different coupling ranges.

*Symmetry properties:* The non-reciprocity term changes the Hamiltonian from Hermitian to non-Hermitian without altering the TRS and PHS. In, non-Hermitian systems, the CS and sub-lattice symmetries are different unlike Hermitian systems^24^. In the current model, the SLS is broken while CS is obeyed (Table 5). In addition to this, the combination of parity and time reversal $\mathscr{P}\mathscr{T}$ symmetry obeyed for the region $t_{ps}>|\delta |$, which gives rise to a real energy spectrum.

**Table 5:** Symmetry operation of a $\mathscr{P}\mathscr{T}$ symmetric non-Hermitian model.

Symmetry	Operation	Result
TRS	$\mathscr {T}H(k)\mathscr {T}^{-1}=H(-k)$	$\checkmark$
PHS	$\mathscr {C}H(k)\mathscr {C}^{-1}=-H(-k)$	$\checkmark$
CS	$\mathscr{T}\mathscr{C}H(k)\mathscr {T}^{-1}\mathscr {C}^{-1}\ne -H(-k)$	X
SLS	$\mathscr {S}H(k)\mathscr {S}^{-1}=-H(k)$	$\checkmark$

Here TRS, PHS, CS and SLS represent time-reversal, particle-hole, chiral and sub-lattice symmetries respectively.

*Complex Berry phase:* Under Hermitian case, the Berry phase is a real quantity and remains quantized under certain symmetries by reflecting the topological order of the system. In case of non-Hermitian systems, the periodic table of symmetry is different and the structure of the topological invariant may not behave similar to that of Hermitian systems. This creates a necessity to generalize the structure of topological invariants to a wide spectrum of non-Hermitian systems, which leads to the concept of complex Berry phase^37–39^. The geometry is constructed in the bi-orthonormal basis, where the structure behaves similar to that of Hermitian Berry phase. The complex Berry phase can be obtained through the Fourier transform of Eq. (40) as

41 $$\begin{aligned} H_{BdG}(k)=\left( \begin{matrix} \chi _z&{}&{}\chi _y\\ \\ -\chi _y&{}&{}-\chi _z \end{matrix} \right) .\end{aligned}$$

with

$$\begin{aligned} \chi _z= & {} -\epsilon _s-\sum _{l=1}^{r}\frac{2t}{l^{\alpha }}\cos (kl),\hspace{0.5cm} \chi _y=\sum _{l=1}^{r}2i\left( \frac{t_{ps}}{l^{\alpha }}\sin (kl)+\frac{\delta }{l^{\alpha }}\sin (kl)\right) \end{aligned}$$

The basis of the Bloch Hamiltonian can be rotated through the unitary generator $\textbf{U}(n)$. The Hamiltonian in new basis can be written as

42 $$\begin{aligned} \left( \begin{matrix} \chi ^{'}_z&{}&{}\chi ^{'}_x+\chi ^{'}_y\\ \chi ^{'}_x-\chi ^{'}_y&{}&{}-\chi ^{'}_z\\ \end{matrix} \right) \end{aligned}$$

where $\chi ^{'}_x,\chi ^{'}_y$ and $\chi ^{'}_z$ are the modified winding vectors. The energy dispersion is given by

43 $$\begin{aligned} E= & {} \sqrt{(\chi ^{'}_x)^2+(\chi ^{'}_y)^2+(\chi ^{'}_z)^2} \end{aligned}$$

The dispersion remains real for the $\mathscr{P}\mathscr{T}$ symmetric regime, where we consider the spectra to calculate the critical exponents. For the $\mathscr{P}\mathscr{T}$ broken regime, the spectra become complex, and we consider the absolute spectra to calculate the critical exponents. Sometimes, the real spectra produces different critical exponents around the single gap closing point, based on the symmetry behavior. To avoid such ambiguity, we consider the absolute spectra which may produce different critical exponents at some parameter space^34^. To understand the geometric phase and associated critical exponents, we introduce bi-orthonormal basis vectors as^24^

44 $$\begin{aligned} |\lambda _{\pm }(k)\rangle =\pm \frac{1}{\sqrt{2}}\beta _1e^{\pm i\kappa ^*}\left( \frac{\sin (\theta _k^*)e^{-i\phi }}{\pm 1+\cos (\theta ^*)} \right) ,\hspace{0.5cm} |\psi _{\pm }(k)\rangle =\pm \frac{1}{\sqrt{2}}\beta _2e^{\pm i\kappa }\left( \frac{\sin (\theta _k)e^{-i\phi }}{\pm 1-\cos (\theta ^*)} \right) \end{aligned}$$

Here $\kappa$ is independent of *k* with

45 $$\begin{aligned} \phi= & {} \tan ^{-1}\left( \frac{-\chi _y}{\chi _z} \right) \hspace{0.5cm} \theta =\tan ^{-1}\left( \frac{\sqrt{\chi _y^2+\chi _x^2}}{\chi _z} \right) \end{aligned}$$

with $\beta _1^*\beta _2=\frac{1}{2}(1-\cos (\theta ))$ which are periodic in *k*. At exceptional point we get $\langle \lambda _{\pm }(k)|\psi _{pm}(k)\rangle =0$. The complex Berry phase is given by^37,40,41^

46 $$\begin{aligned} W_{\pm }= & {} i\oint _{BZ}\langle \lambda _{\pm }(k)|\bigtriangledown _k|\psi _{\pm }(k)\rangle dk =\frac{1}{2}\oint _{BZ}\frac{\partial \phi }{\partial k}\left( 1+\cos (\theta )\right) dk. \end{aligned}$$

Complex Berry phase gives the Hermitian equivalence of the geometric phase by deploying the left and right eigenvectors. The integrand of Eq. (46) can be used as a curvature function to determine critical exponents. Here, we consider three different ranges of couplings to understand the behavior of the system. Due to the structure of energy dispersion (Eq. 43), for each range of couplings, the criticality condition remains similar to that of their Hermitian counterparts. Hence the phase diagram remains similar in Hermitian and $\mathscr{P}\mathscr{T}$ symmetric models, but the symmetry behavior changes for different parameter spaces.

#### Short-range

Here, the winding vectors in rotated basis is given by,

$$\begin{aligned} \chi ^{'}_x= & {} -\epsilon -2t(\cos (k)),\hspace{0.5cm} \chi ^{'}_y=-2t_{ps}(\sin (k))\hspace{0.5cm} \chi ^{'}_z=-2i\delta (\sin (k)) \end{aligned}$$

The energy dispersion and complex Berry phase is given by

47 $$\begin{aligned} E= & {} \sqrt{\left( -\epsilon -2t \cos (k) \right) ^2+4(t_{ps}^2-\delta ^2)\left( \sin (k)\right) ^2}\nonumber \\ W= & {} \frac{1}{2}\oint _{BZ}\frac{(2 t_{ps} (\epsilon \cos (k)-2 t)) }{4 t_{ps} ^2 \sin ^2(k)+(2 t \cos (k)-\epsilon )^2}. \left( 1+\frac{2 i \delta \sin (k)}{\sqrt{4 \left( t_{ps} ^2-\delta ^2\right) \sin ^2(k)+(2 t \cos (k)-\epsilon )^2}}\right) dk \end{aligned}$$

where the spectrum preserves $\mathscr{P}\mathscr{T}$ symmetry for $t>\delta$. At $t=\delta$ there occurs a transition from $\mathscr{P}\mathscr{T}$ symmetric to $\mathscr{P}\mathscr{T}$ broken phase and the spectrum becomes complex for $\delta >t$. The universality class of critical exponents remain similar to that of Hermitian system under $\mathscr{P}\mathscr{T}$ symmetry, and we consider the $\mathscr{P}\mathscr{T}$ broken phase and $\mathscr{P}\mathscr{T}$ transition point for critical analysis.

**Figure 8:**
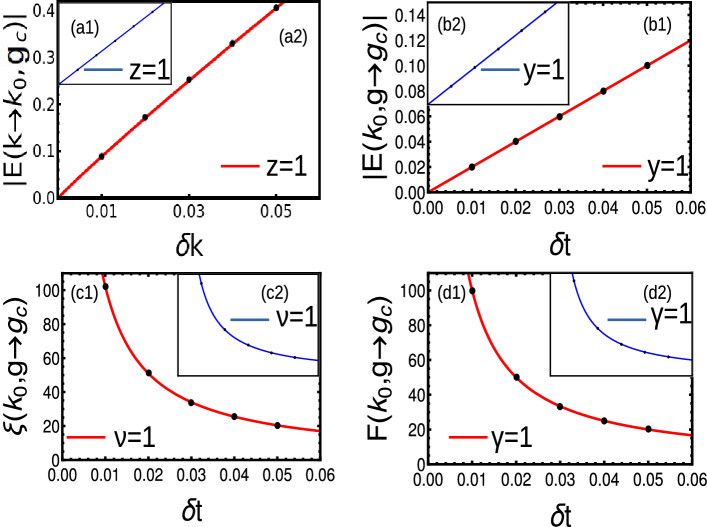
(**a**1–**d**1) Dynamic, crossover, localization and susceptibility critical exponents of short-range Hamiltonian at $\mathscr{P}\mathscr{T}$ symmetry breaking point. (**a**2–**d**2) The insets represent the same critical exponents with absolute spectra in the $\mathscr{P}\mathscr{T}$ broken regime.

At $t_{ps}=\delta$, the dispersion becomes linear and the crossover exponent shows a linear fitting. The exponents $\gamma$ and $\nu$ can be calculated through Eqs. (54) and (54) respectively, which yield linear fittings (Fig. 8a1–d1). For the value $t_{ps}<\delta$ the Hamiltonian becomes $\mathscr{P}\mathscr{T}$ broken producing the non-Hermitian skin effect. The energy spectrum yields the complex values, where the exponents may differ for real and absolute spectra. Here we consider only absolute spectrum and get the universality class $z=y=\nu =\gamma =1$ respectively (Fig. 8a2–d2).

#### Extended-range

For the case $r=2$, we get winding vectors in rotated basis as,

$$\begin{aligned} \chi ^{'}_x= & {} -\epsilon -2t(\cos (k)+\frac{\cos (2k)}{2^{\alpha }}),\hspace{0.5cm} \chi ^{'}_y=-2t_{ps}(\sin (k)+\frac{\sin (2k)}{2^{\alpha }})\hspace{0.5cm} \chi ^{'}_z=-2i\delta (\sin (k)+\frac{\sin (2k)}{2^{\alpha }}) \end{aligned}$$

The energy dispersion and complex Berry phase are given by

48 $$\begin{aligned} E= & {} \sqrt{\left( \epsilon +2t\left( \cos (k)+\frac{\cos (2k)}{2^{\alpha }}\right) \right) ^2+ 4(t^2-\delta ^2)\left( \sin (k)+\frac{\sin (2k)}{2^{\alpha }}\right) ^2}\nonumber \\ W= & {} \frac{1}{2}\oint _{BZ}\frac{(2 t_{ps} (\epsilon (\cos (k)+\cos (2k)/2^{\alpha })-2 t)) }{4 t_{ps} ^2 (\sin (k)+\sin (2k)/2^{\alpha })+(2 t (\cos (k)+\cos (2k)/2^{\alpha })-\epsilon )^2}.\nonumber \\{} & {} \left( 1+\frac{2 i \delta (\sin (k)+\sin (2k)/2^{\alpha })}{\sqrt{4 \left( t_{ps} ^2-\delta ^2\right) (\sin (k)+\sin (2k)/2^{\alpha })^2+(2 t (\cos (k)+\cos (2k)/2^{\alpha })-\epsilon )^2}}\right) dk \end{aligned}$$

**Figure 9:**
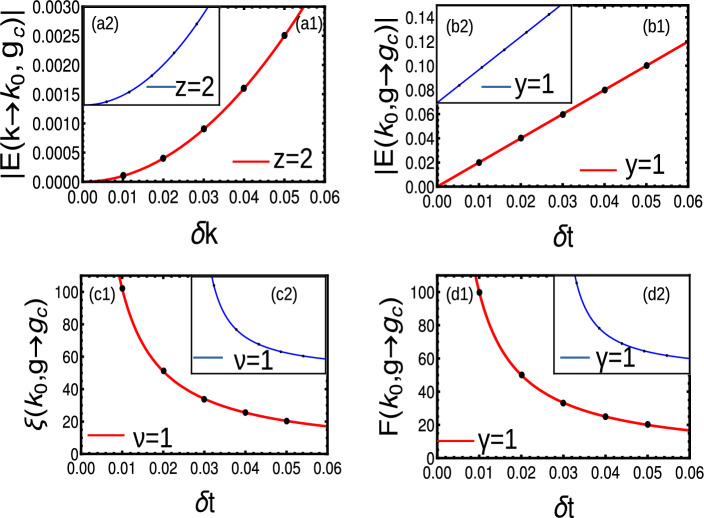
(**a**) (**a**1–**d**1) Dynamic, crossover, localization and susceptibility critical exponents of short-range Hamiltonian at $\mathscr{P}\mathscr{T}$ symmetry breaking point. (**a**2–**d**2) The insets represent the same critical exponents with absolute spectra in the $\mathscr{P}\mathscr{T}$ broken regime.

The universality class of critical exponents yield $z=y=\nu =\gamma =1$ for all criticalities except at multi-critical point (not shown here). At multi-critical point, $z=2$ at $t_{ps}=\delta$ where the $\mathscr{P}\mathscr{T}$ symmetry breaks. Interestingly, $\nu =1$ calculated from Eq. (54), resulting in the violation of $z\nu =1$ scaling relation (Fig. 9 a1–d1). For $t_{ps}<\delta$, we consider absolute spectrum $z=2$ and $y=\nu =\gamma =1$ (Fig. 9 a2–d2).

#### Long-range

Here, the winding vectors in rotated basis is given by,

$$\begin{aligned} \chi ^{'}_x= & {} -\epsilon -2t(\frac{Li_{\alpha }[e^{ik}]+Li_{\alpha }[e^{-ik}]}{2}),\hspace{0.5cm} \chi ^{'}_y=-2t_{ps}(\frac{Li_{\alpha }[e^{ik}]-Li_{\alpha }[e^{-ik}]}{2i})\hspace{0.5cm} \chi ^{'}_z=-2i\delta (\frac{Li_{\alpha }[e^{ik}]-Li_{\alpha }[e^{-ik}]}{2i}) \end{aligned}$$

The energy dispersion and complex Berry phase is given by

49 $$\begin{aligned} E= & {} \sqrt{\left( -\epsilon -2t \frac{Li_{\alpha }[e^{ik}]+Li_{\alpha }[e^{-ik}]}{2} \right) ^2+4(t_{ps}^2-\delta ^2)\left( \frac{Li_{\alpha }[e^{ik}]-Li_{\alpha }[e^{-ik}]}{2i}\right) ^2}\nonumber \\ W= & {} \frac{1}{2}\oint _{BZ}\frac{(2 t_{ps} (-\epsilon \frac{Li_{\alpha }[e^{ik}]+Li_{\alpha }[e^{-ik}]}{2}-2 t)) }{4 t_{ps} ^2 (\frac{Li_{\alpha }[e^{ik}]-Li_{\alpha }[e^{-ik}]}{2i}) ^2+(2 t \frac{Li_{\alpha }[e^{ik}]+Li_{\alpha }[e^{-ik}]}{2}-\epsilon )^2}.\nonumber \\{} & {} \left( 1+\frac{2 i \delta \frac{Li_{\alpha }[e^{ik}]-Li_{\alpha }[e^{-ik}]}{2i}}{\sqrt{4 \left( t_{ps} ^2-\delta ^2\right) (\frac{Li_{\alpha }[e^{ik}]-Li_{\alpha }[e^{-ik}]}{2i})^2+(2 t \frac{Li_{\alpha }[e^{ik}+Li_{\alpha }[e^{-ik}]}{2}-\epsilon )^2}}\right) dk \end{aligned}$$

**Figure 10:**
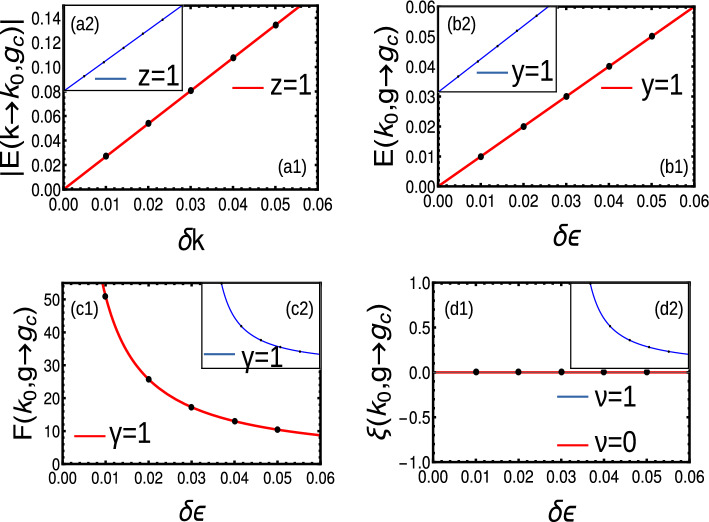
Dynamic, crossover, localization and susceptibility critical exponents of long-range Hamiltonian in the regime $\alpha >2$ (**a**1–**d**1) at $\mathscr{P}\mathscr{T}$ symmetry breaking point. (**a**2–**d**2) The insets represent the same critical exponents with absolute spectra in the $\mathscr{P}\mathscr{T}$ broken regime.

For $t_{ps}<\delta$, the universality class of critical exponents yield $z=y=\nu =\gamma =1$ in the regime $\alpha >2$. Here we consider absolute spectrum, which results in the domination of non-Hermitian properties (Fig. 10a2–d2). At the point of $\mathscr{P}\mathscr{T}$ symmetry breaking ($t_{ps}=\delta$), the localization exponent vanishes as the localization length becomes slop less (Fig. 10a1–d1). In the regime $1<\alpha <2$, the dynamical exponent yields fractional values with crossover exponent $y=1$. The susceptibility and localization exponents are ill-defined in this regime (not shown here). A comparison of critical exponents are presented in Table 6

**Table 6:** A comparison of universality class of critical exponents between short, extended and long-range of $\mathscr{P}\mathscr{T}$ symmetric non-Hermitian model for different regimes of parameters.

	$\mathscr{P}\mathscr{T}$ breaking point				$\mathscr{P}\mathscr{T}$ broken regime			
	z	y	$\nu$	$\gamma$	z	y	$\nu$	$\gamma$
Short-range	1	1	1	1	1	1	1	1
Extended-range	1	1	1	1	2	1	1	1
(normal criticalities)								
Extended-range	2	1	1	1	2	1	1	1
(multi-criticalities)								
Long-range	1	1	IL	1	1	1	IL	1
($$\alpha >2$$)								

Here IL and D represent ill-defined and divergent quantities.

## Discussion

### Manifestation of Lorentz invariance at multi-critical points

As per the scaling law, the linear dispersion of energy (at gap closing point/Dirac points) always preserves the Lorentz invariance . Dynamical critical exponent (*z*) is the quantity which reflects the zero mass condition, including the information about the Lorentz invariance^42^. At Fermi surface, the energy dispersion is similar to relativistic form (which is a Lorentz invariant), i.e., $E^2=c^2p^2+m^2c^4$, which can reduce to massless case (under criticality). Hence, it becomes a linear equation which connects the energy and momentum ($E^2=c^2p^2$).

- In longer-range model, due to the multi-criticality effect, the dispersion relation acquires some correction term i.e., $E^2=c^2p^2+m^2c^4+Ap^4$, where *A* is a constant^43^. This results in the violation of Lorentz invariance and gives rise to quadratic dispersion of energy $z=2$. The velocity of quasi-particles around $k=\pi$ criticality is given by,
  50 $$\begin{aligned} \frac{dE_k}{dk}= & {} \frac{A(-1+\frac{1}{2^{\alpha -1}})^3k^2}{\sqrt{(\frac{B}{2^{\alpha -1}}))^2+(C(-1+\frac{2}{2^{\alpha }})k)^2}}, \end{aligned}$$
  where A, B and C are constants. For criticality, the velocity vanishes as $\textbf{g}\rightarrow \textbf{g}$. But for multi-criticality, the velocity becomes indeterminate as $\alpha \rightarrow 1$.
- In long-range model, the dimensionality of the system may change depending on the strength of coupling^15,16^. In the $1<\alpha <2$ region, the Lorentz invariance breaks and the quasi-particles near the criticality feel the effective dimension. The velocity of quasi-particles around $k=0$ criticality is given by,
  51 $$\begin{aligned} \frac{dE_k}{dk}= & {} \frac{Ak^{2\alpha -3}+Bk^{\alpha -1}+Ck}{\sqrt{A^2k^{2\alpha -2}+Bk^{\alpha }+C^2k^2}}. \end{aligned}$$
  where *A*, *B* and *C* are constants. For the limit $\alpha >2$, the velocity vanishes as $k\rightarrow 0$. When $z=1$, the exited quasi-particles near the gapless states feel a fixed speed. For $z>1$, the speed is not fixed and for $z<1$, the exited quasi-particles possess very small momenta and contains no upper limit to the speed^44,45^.In long-range models, the conservation of Lorentz invariance in the low excitation spectrum validates the correlation exponents through Ornstein-Zernike equations. As the Lorentz invariance is broken in the region $1<\alpha <2$, the CF fails to express the correlation and susceptibility exponents.
- With the broken time reversal symmetry, the Hermitian model produces the real spectrum with different dispersion around single gap closing point. The multi-critical point exhibits the dispersion with a coefficient higher than $z=2$. The $\chi _0$ term contributes majorly to this kind of dispersion and we observe the breaking of Lorentz invariance.
- The $\mathscr{P}\mathscr{T}$ symmetric Hamiltonian preserves the Lorentz invariance except at some multi-criticalities (extended-range) and $1<\alpha <2$ region of a long-range model for the case $t>\delta$. For the values $t<\delta$, the $\mathscr{P}\mathscr{T}$ symmetry breaks and produces the complex spectra with $z=1/2$ (this is for real part, where the absolute spectra produce $z=1$). Thus the system becomes non-Hermitian and shows the signatures of non-Hermitian skin effect. This kind of Hamiltonian naturally breaks the Lorentz invariance including multi-critical point^46^.

There are a number of examples where one can observe the violation of Lorentz invariance like in graphene^47^, 3D Weyl semi-metals^48,49^ and transition from Dirac semi-metal to band insulator^50^. However, in longer-range models, the topological transitions are found to be Lorentz invariant except transitions across some multi-critical points^29,51^.

### Extended-range effects

One of the main motivation of this work is to understand the behavior of criticality with extended-range of coupling parameters.

- In Hermitian systems, with the increasing number of couplings, we observe the staircase of transitions, where the uppermost winding number will be directly related to the number of coupling neighbor^17^. For the even (odd) number of neighbors ($r>2$), we obtain the staircase of transitions occurs only among even (odd) winding numbers. The formation of multi-criticality occurs only during the even neighbors, where we observe the breaking of Lorentz invariance. The transition among odd winding numbers resembles the universality class of that of short-range models.
- With broken TRS, the model do not shows any staircase of transitions. In fact, there occurs only two regions namely gapped and gapless, where there are no proper tools to characterize the topological invariant. The multi-criticality can occur irrespective of the number of neighbors and the formation of multi-criticality depends on the value of $\phi$. This multi-criticality is different that the normal criticality and shows the Lorentz violations.
- With $\mathscr{P}\mathscr{T}$ symmetric and $\mathscr{P}\mathscr{T}$ broken models, we observe staircase of transitions similar to Hermitian case, where the multi-criticalities show the violation of Lorentz invariance.

## Method

### Analytical calculation of critical exponents

The quasi-particle excitation energy is given by

52 $$\begin{aligned} E(k)=\pm \sqrt{(\chi _z(k))^2+(\chi _y(k))^2}=\sqrt{|\partial g|^{2\nu z}+k^{2z}}. \end{aligned}$$

where $\partial g=t-t_c$, which is the distance to the criticality^28,29^. With the gap closing ($t=t_c$), the edge mode decays into the bulk with $\xi \propto |g|^{-\nu }$, where $\xi$ is the correlation length and $\nu$ is the corresponding critical exponent. At $t=t_c$ (QCP), $E_k\propto k^z$ where *z* is the dynamical critical exponent, which determines the nature of energy dispersion.

The expansion of pseudo-spin vector around the QCP at $k=k_0$ gives the nature of dispersion. i.e.,

53 $$\begin{aligned} \chi (k_0)=\chi (k_0)+\chi ^{\prime }(k_0)k+\chi ^{\prime \prime }(k_0)k^2/2. \end{aligned}$$

Plugging this into the energy dispersion in Eq. (52),

54 $$\begin{aligned} E_k= & {} \sqrt{(\delta g+Ck^2)^2+(Dk)^2}\nonumber \\= & {} \sqrt{\delta g+A_4k^4+A_2k^2}, \end{aligned}$$

where $A_4=C^2$ and $A_2=2\delta gC+D^2$. The coefficient of $k^2$ and $k^4$ are responsible for linear and quadratic dispersion respectively. The dominant term among $A_4$ and $A_2$ decides the nature of dispersion, which gives the dynamical critical exponent *z*.

Correlation critical exponent can be calculated through the Eq. (14), we get Ornstein-Zernike form. i.e.,

55 $$\begin{aligned} F(k,M)\mid _{k=k_0}= & {} \frac{A.\delta k(2B.\delta k)-(\delta g+B\delta k^2)A}{\delta g^2+(2B\delta g+A^2)\delta k^2+B^2\delta k^4}\nonumber \\= & {} \frac{\frac{2AB\delta k^2-A(\delta g+B\delta k^2)}{\delta g^2}}{1+\left( \frac{2\delta g.B+A^2}{\delta g^2} \right) \delta k^2+\left( \frac{B^2}{\delta g^2} \right) \delta k^4 }\nonumber \\= & {} \frac{F(k_0,\delta g)}{1+\xi ^2\delta k^2+\xi ^4\delta k^4}, \end{aligned}$$

where $\xi$ is the correlation function. In Eq. (55), there are two terms which decides the correlation function. (1) $\xi \propto \sqrt{\frac{2B}{\delta g}+\frac{A^2}{\delta g^2}}$, where the term $\frac{A^2}{\delta g^2}$ dominates over $\frac{2B}{\delta g}$. Hence $\xi \propto 1/|\delta g|\Rightarrow \nu =1$. (2) $\xi \propto \root 4 \of {\frac{B^2}{\delta g^2}}$ and $\xi \propto 1|\delta g|^{-1/2}$. Thus the dominating term among *A* and *B* decides the correlation critical exponent.

**Table 7:** Table of critical exponents for longer-range two orbital model with $t=0.5$ obtained by an analysis of Eqs. (14) and (7).

*r*	$\hspace{0.13cm}\alpha \hspace{0.13cm}$	$\epsilon$	$(|A_2|,|A_4|)$	(|*A*|, |*B*|)	$k_0=0$	$k_0=\pi$
		$(k_0=0,\pi )$	$(k_0=0,\pi )$	$(k_0=0,\pi )$	$(z,\nu )$	$(z,\nu )$
2	0	($-$ 2,0)	(36,–)(4,–)	(600,–)(200,–)	(1,1)	(1,1)
	0.1	($-$ 1.93,0.06)	(32.8573,–)(3.0003,–)	(944.963,–)(440.296,–)	(1,1)	(1,1)
	0.5	($-$ 1.70,0.29)	(23.3137,–)(0.6863,–)	(1145.93,–)(143.167,–)	(1,1)	(1,1)
	0.9	($-$ 1.53,0.46)	(17.1690,–)(0.0206,–)	(2336.42,–)(17.4493,–)	(1,1)	(1,1)
	1	($-$ 1.5,0.5)	(16,–)(0,4.12)	(400,–)(0,3.81)	(1,1)	(2,1/2)
	1.1	($-$ 1.46,0.53)	(14.9465,–)(0.0179,–)	(1274.67,–)(19.224,–)	(1,1)	(1,1)
	1.4	($-$ 1.37,0.62)	(12.3603,–)(0.2345,–)	(447.39,–)(226.119,–)	(1,1)	(1,1)
	1.9	($-$ 1.26,0.73)	(9.4358,–)(0.8616,–)	(521.813,–)(225.666,–)	(1,1)	(1,1)
3	0	($-$ 3,1)	(144,–)(16,–)	(1200,–)(400,–)	(1,1)	(1,1)
	0.1	($-$ 2.82,0.96)	(123.3850,–)(13.2760,–)	(1391.46,–)(622.741,–)	(1,1)	(1,1)
	0.5	($-$ 2.28,0.87)	(68.7660,–)(6.9468,–)	(930.271,–)(5412.33,–)	(1,1)	(1,1)
	0.9	($-$ 1.90,0.8361)	(40.6507,–)(4.3627,–)	(1088.84,–)(904.726,–)	(1,1)	(1,1)
	1	($-$ 1.83,0.8333)	(36,–)(4,–)	(489.273,–)(358.8,–)	(1,1)	(1,1)
	1.1	($-$ 1.76,0.8321)	(32.0128,–)(3.7089,–)	(16708.4,–)(450.739,–)	(1,1)	(1,1)
	1.4	($-$ 1.59,0.8358)	(23.0833,–)(3.1438,–)	(644.529,–)(1020.34,–)	(1,1)	(1,1)
	1.9	($-$ 1.39,0.8561)	(14.5608,–)(2.7966,–)	(974.899,–)(781.219,–)	(1,1)	(1,1)

Throughout the analysis, with the even number of neighbors, 1st TQCL $k=0$ exhibits same universality class i.e., $(z=1, \nu =1)$. But 2nd TQCL $k=\pi$ shows a breakdown at $\alpha =1$ i.e., $(z=1, \nu =1)$ to $(z=2,\nu =1/2)$. For the odd interacting neighbors, the universality class remains same for both $k=0$ and $k=\pi$.

Through Table 7, we observe that, the quadratic dispersion occurs only for the systems with even number of couplings. This observation holds true for higher number of couplings also.

## Conclusion

Multi-criticalities in the longer-range (finite neighbors) models are the combinations of criticalities of different nature. The localization property and the behavior of topological invariant at criticality ignites the curiosity in this area. This kind of multi-criticality also witness different possible transitions which can also be recognized by the universality class of critical exponents. It also helps to categorize the criticalities and to recognize the breaking of Lorentz invariance. The behavior is consistent with multi-criticalities with different symmetry behaviors. At some stage, multi-criticalities in an extended model with broken time-reversal symmetry exhibits an exponent with $z=4$, which is similar to the flat band structures. The methodology is extended to long-range models (infinite neighbors), where the universality class of critical exponents recognizes $1<\alpha <2$ as Lorentz violated region and $\alpha =2$ as short-range limit. To summarize, we present a theoretical study of criticality for a short, extended and long-range topological chain under different symmetry conditions. Instances of interplay of criticality and extended-range under symmetry constrains are rare in literature, and we hope our work will be interesting in understanding such systems.

## Data Availability

The datasets used and/or analyzed during the current study available from the corresponding author on reasonable request.
